# The Singular Evolution of *Olea* Genome Structure

**DOI:** 10.3389/fpls.2022.869048

**Published:** 2022-03-31

**Authors:** Flavia Mascagni, Elena Barghini, Marilena Ceccarelli, Luciana Baldoni, Carlos Trapero, Concepción Muñoz Díez, Lucia Natali, Andrea Cavallini, Tommaso Giordani

**Affiliations:** ^1^Department of Agriculture, Food and Environment, University of Pisa, Pisa, Italy; ^2^Department of Chemistry, Biology and Biotechnology, University of Perugia, Perugia, Italy; ^3^CNR, Institute of Biosciences and BioResources, Perugia, Italy; ^4^CSIRO Agriculture & Food, Narrabri, NSW, Australia; ^5^Agronomy Department, University of Cordoba, Cordoba, Spain

**Keywords:** *Olea* evolution, tandem repeats, retrotransposons, genome landscape, NGS analyses, genome evolution

## Abstract

The current view of plant genome evolution proposes that genome size has mainly been determined by polyploidisation and amplification/loss of transposons, with a minor role played by other repeated sequences, such as tandem repeats. In cultivated olive (*Olea europaea* subsp. *europaea* var. *europaea*), available data suggest a singular model of genome evolution, in which a massive expansion of tandem-repeated sequences accompanied changes in nuclear architecture. This peculiar scenario highlights the importance of focusing on *Olea* genus evolution, to shed light on mechanisms that led to its present genomic structure. Next-generation sequencing technologies, bioinformatics and *in situ* hybridisation were applied to study the genomic structure of five related *Olea* taxa, which originated at different times from their last common ancestor. On average, repetitive DNA in the *Olea* taxa ranged from ~59% to ~73% of the total genome, showing remarkable differences in terms of composition. Among repeats, we identified 11 major families of tandem repeats, with different abundances in the analysed taxa, five of which were novel discoveries. Interestingly, overall tandem repeat abundance was inversely correlated to that of retrotransposons. This trend might imply a competition in the proliferation of these repeat classes. Indeed, *O. paniculata*, the species closest to the *Olea* common ancestor, showed very few tandem-repeated sequences, while it was rich in long terminal repeat retrotransposons, suggesting that the amplification of tandem repeats occurred after its divergence from the *Olea* ancestor. Furthermore, some tandem repeats were physically localised in closely related *O. europaea* subspecies (i.e., cultivated olive and *O. europaea* subsp. *cuspidata)*, which showed a significant difference in tandem repeats abundance. For 4 tandem repeats families, a similar number of hybridisation signals were observed in both subspecies, apparently indicating that, after their dissemination throughout the olive genome, these tandem repeats families differentially amplified maintaining the same positions in each genome. Overall, our research identified the temporal dynamics shaping genome structure during *Olea* speciation, which represented a singular model of genome evolution in higher plants.

## Introduction

The current view of plant genome evolution proposes that genome size is determined by processes such as polyploidisation and amplification/loss of transposable elements (TEs), especially retrotransposons (REs; Proost et al., 2011; Catlin and Josephs, 2022). The genome of most plant clades has been shaped during evolution by many polyploidisation events, with each new episode superimposed on genomic remnants from earlier rounds of duplication. At the same time, the bulk of non-coding DNA in plant genomes consists of active, silenced or degenerating mobile elements, which vary widely in composition and abundance among populations (Garrido-Ramos, 2015; Wendel et al., 2018).

Mobile elements can affect genomes either during mobilisation events or after their insertion. Mobilisation of a TE and its insertion within the coding sequence of a gene, or nearby the promoter, can lead to a loss of function or altered expression of that gene (Dubin et al., 2018). Furthermore, TE proliferation, or loss, produces changes in genome size. Notable examples are *Oryza australiensis*, where amplification of specific retrotransposon lineages has led to the doubling of its genome size within the last 3 million years (Piegu et al., 2006), and the legume tribe *Fabeae*, where genome dynamics, are dominated by a single lineage of REs that accounts for 57% of the variation in genome size in this clade (Macas et al., 2015). The impact of TEs on the genomic landscape continues after insertion, contributing to the organisation of the genome through epigenetic regulation (Lippman et al., 2004; Hollister and Gaut, 2009; Usai et al., 2021), or by still affecting gene expression after becoming transcriptionally inactive (Marcon et al., 2015; Sanseverino et al., 2015).

Transposable elements are classified into two different classes, according to whether their transposition intermediate is RNA (Class I or REs) or DNA (Class II or DNA transposons; Wicker et al., 2007). In plants, REs are the most common class of elements, representing the core of many genomes (Lisch, 2013; Vitte et al., 2014), and are further classified into five taxonomic orders (Wicker et al., 2007). The most abundant REs in plants, long terminal repeat retrotransposons (LTR-REs), are organised into two major superfamilies, called *Gypsy* and *Copia*, which differ in the position of protein domains within their encoded polyprotein (Wicker et al., 2007). In turn, the superfamilies can be classified into several major evolutionary lineages (Wicker and Keller, 2007; Llorens et al., 2010), seven lineages for *Copia* and three main lineages for *Gypsy* (Buti et al., 2017; Neumann et al., 2019; Vangelisti et al., 2019; Mascagni et al., 2020).

Other types of repeated sequences generally have a minor role in shaping plant genome structure and size, accounting for a small portion of the genomes sequenced so far. Among these, tandem repeats (TRs) are arranged in tandem repeating units, where individual copies lie adjacent to one another, and usually show different GC content from the rest of the genomic DNA (Szybalski, 1968).

Precise molecular mechanisms leading to TR proliferation in individual species and/or to their rapid turnover have not yet been clearly identified. Several mechanisms have been proposed for the generation of short arrays of TRs, including unequal crossing over of random sequences (Smith, 1976), slipped-strand mispairing (Levinson and Gutman, 1987) and sequence-directed mutagenesis (Fieldhouse and Golding, 1991). In addition, tandem duplications of varying length can also result from aberrant replication and replication stress (Mazurczyk and Rybaczek, 2015; Nikolov and Taddei, 2016).

Initially isolated from satellite bands in gradient centrifugation experiments, TRs are commonly known as satellite DNA (Schmidt and Heslop-Harrison, 1998). Satellite arrays are generally found in heterochromatic regions and may form essential chromosome structures such as centromeres and telomeres (Garrido-Ramos, 2017; Hartley and O’Neill, 2019). Apart from their common key role in these critical structures, TR families are characterised by a huge variety of sequences (Melters et al., 2013) differing in location, repeat unit length and abundance, suggesting they undergo rapid evolution (Thakur et al., 2021). Being one of the most dynamic components of eukaryotic genomes, most satellite repeat families are usually species- or genus-specific (Garrido-Ramos, 2015).

On the other hand, evidence of sequence conservation of satellite families for long evolutionary periods among species has also been reported (Quesada del Bosque et al., 2013, 2014; Cafasso and Chinali, 2014; Mehrotra et al., 2014), supporting the hypothesis of a possible functional role for these sequences in the genomes (Pezer et al., 2012; Plohl et al., 2012). Therefore, related species may share an ancestral set of satellite families with specific levels of conservation and amplification.

In the cultivated olive (*Olea europaea* subsp. *europaea* var. *europaea*), available data suggest a singular model of genome evolution, in which polyploidisation and amplification/loss of TEs were accompanied by a massive expansion of the tandemly repeated fraction. As a result, TRs compose almost one-third of the current olive genome, a much larger portion than in the vast majority of plant genomes (Barghini et al., 2014).

Several studies were conducted to elucidate the TR fraction of olive, with six TR families being isolated from genomic libraries, and in some case, localised by cytological hybridisation (Katsiotis et al., 1998; Bitonti et al., 1999; Minelli et al., 2000; Lorite et al., 2001; Contento et al., 2002 Barghini et al., 2014).

A first genome sequence for *Olea europaea* subsp. *europaea* var. Farga was released in 2016 (Cruz et al., 2016) with a limited characterisation of the repeated component; then, a genome sequence and annotation of the wild olive tree (*Olea europaea* subsp. *europaea* var. *sylvestris*; Unver et al., 2017) resulted in contrast with previous studies showing a significantly lower abundance of TRs than expected. The most recent studies related to the genome of cultivated olive, although revealed a great genetic variability as result of a significant activation of TEs during the domestication process (Jiménez-Ruiz et al., 2020), made only little progress in deciphering the complex structure of its repetitive component (Rao et al., 2021).

The difficulty in identifying satellite sequences might be explained by repeat collapse, which causes common mis-assembly due to the incorrect gauging of the number of repeat copies in a genome, and ultimately providing a reference with too few repeat copies (Phillippy et al., 2008).

New possibilities for investigating repetitive sequences in genomes were provided by massive parallel DNA sequencing techniques. In fact, the use of these technologies within a computational framework led to the identification of the different types of repetitive elements, allowing us to address many features of the dynamics which have changed the repetitive component of the *Olea* genome.

In this study, we aimed at characterising the repetitive component of a range of taxa representative of the *Olea* genus, including plants from different geographical origins. We also included *O. paniculata* as representative species of the subgenus *Paniculatae*, the closest relative of the *Olea* last common ancestor. This analysis represents the most comprehensive study of the evolutionary dynamics of repetitive elements within *Olea* genus, evaluating with different methodologies (bioinformatic, cytophotometric and cytological) how the genome structure has evolved and shedding light on mechanisms of genome expansion.

## Materials and Methods

### Plant Material, DNA Isolation, and Illumina Sequencing

For this study, the following species of *Olea* were chosen, *O. paniculata*, a representative of the subgenus *Paniculatae*, and four taxa of the subgenus *Olea*, *O. exasperata* (section *Ligustroides*), *O. europaea* subsp. *europaea* (cv. Leccino), *O. europaea* subsp. *cuspidata* and *O. europaea* subsp. *guanchica* (Table 1). Plant material (leaves and root apices, the latters collected from potted plants or cuttings) was provided by the Olive Collection of CNR—Institute of Biosciences and Bioresources, Division of Perugia (Perugia, Italy), by the IFAPA World Olive Germplasm Bank and Agronomy Department of University of Cordoba (Cordoba, Spain) and by CSIRO Agriculture & Food (Narrabri, NSW, Australia).

**Table 1:** *Olea* taxa analysed and number of Illumina reads used for the analyses.

Subgenus	Section	Species	Subspecies	Origin	Sample source	Raw reads	Trimmed reads
*Olea*	*Olea*	*O. europaea*	*europaea*	Italy	CNR-IBBR^1^	71,624,494	47,023,392
*Olea*	*Olea*	*O. europaea*	*guanchica*	Canary Islands	IFAPA^2^	16,457,568	13,478,858
*Olea*	*Olea*	*O. europaea*	*cuspidata*	Ethiopia	IFAPA^2^	20,368,004	16,175,030
*Olea*	*Ligustroides*	*O. exasperata*	–	South Africa	IFAPA^2^	15,348,186	12,211,284
*Paniculatae*	*-*	*O. paniculata*	–	Australia	CSIRO^3^	20,622,182	17,243,520

1 *Olive Collection of CNR—Institute of Biosciences and Bioresources, Division of Perugia (Perugia, Italy).*

2 *IFAPA World Olive Germplasm Bank (Cordoba, Spain).*

3 *CSIRO Agriculture & Food (Narrabri, NSW, Australia)*.

Genomic DNA was extracted from young leaves using a GenElute Plant Genomic DNA Miniprep kit (Sigma-Aldrich) and following the manufacturer’s instructions. Paired-end libraries were prepared as recommended by Illumina Inc. (San Diego, CA), with minor modifications, and sequencing was performed for all taxa samples.

Whole-genome shotgun sequences described are available on NCBI Sequence Read Archive under the accession number SRX465835 (*O. europaea* subsp. *europaea* cv. Leccino) and BioProject PRJNA810942 for the other analysed taxa.

Paired reads were first tested for quality and trimmed at 100 nt in length, using Trimmomatic (Bolger et al., 2014) with the parameters, leading:20 trailing:20 slidingwindow:4:20 crop:100 minlen:100. Duplicated reads and those containing organelle DNA sequences were removed using CLC-BIO Genomic Workbench 9.5.3 (CLC-BIO, Aarhus, Denmark).

### Repeat Characterisation From NGS Reads

In order to perform a comparative analysis of the repetitive components of five taxa of the genus *Olea*, RepeatExplorer (Novák et al., 2013), a sequence similarity-based clustering method was applied allowing *de novo* identification of repeats and an estimation of their proportion in each genome. A random set of 1,500,000 sequences was used for each species, and these were analysed individually to maximise the number of analysed reads and the sensitivity and accuracy of the repeat data obtained allowing the identification of less abundant repeat families. Because of the large amount of satellite DNA sequence recovered by the software, after preliminary analysis, a filtering of abundant satellite repeats was performed. Using custom libraries, we filtered large satellite repeats from our data to allow more reads to be analysed during repeat identification.

RepeatExplorer output was parsed to collect the clusters identified as repeats. To increase the number of annotated clusters, similarity searches on the remaining unknown clusters were performed by BLASTN and tBLASTX against a library of 254 putative full-length REs of olive (Barghini et al., 2014).

Putative satellite repeats identified *via* graph-based clustering by RepeatExplorer were collected for each species. The validation of monomer sequences of selected satellites was performed by dot plot analysis of the contigs assembled and by using tandem repeat finder (Benson, 1999) and CAP3 (Huang and Madan, 1999) tools.

TR sequences were collected per species and the database was cleaned of redundant sequences by using CD-HIT (Li and Godzik, 2006) with a threshold identity of 95%. A subset of unique sequences was also obtained after grouping the entire collection of TRs.

### Mapping Procedure for Abundance Estimation

Abundance values of sequences were estimated for each taxon by counting the number of reads mapping into clusters of interspersed repeated sequences or into the library of tandem repeat sequences, per million total reads. This method had already been used for many plant species (Swaminathan et al., 2007; Tenaillon et al., 2011; Natali et al., 2013; Mascagni et al., 2015, 2017a, 2018a) including olive (Barghini et al., 2014, 2015). CLC-BIO Genomic Workbench was used to perform mapping with the following parameters: mismatch cost = 1, deletion cost = 1, insertion cost = 1, similarity = 0.7 and length fraction = 0.7.

### Phylogenetic Trees

A multiple sequence alignment of the TR sequences was performed using Clustal Omega (McWilliam et al., 2013), and phylogenetic trees were built using a neighbour joining clustering method (NJ; 1,000 bootstrap replications).

A dendrogram, based on the genome proportions, using data of each isolated TR, was built by using the R package pvclust version 1.3–2 (Suzuki and Shimodaira, 2006), which allowed the assignment of the uncertainty in hierarchical cluster analysis *via* multiscale bootstrap resampling with 10,000 bootstrap replications.

### RE Insertion Time Analysis

Domain-based ANnotation of Transposable Elements (DANTE) was used to identify and extract conserved regions of reverse transcriptase (RT) protein domains for *Gypsy* and *Copia* RE superfamilies. Timing of LTR-REs proliferation bursts of the analysed species was measured according to Piegu et al. (2006) Buti et al. (2011) and Mascagni et al. (2017b, 2018b), through analysis of the distribution of divergence values between pairwise comparisons of sequences belonging to the same lineage. After collecting all RT domain-related sequences from RepeatExplorer results, cluster mapping was performed using CLC-BIO Genomic Workbench to isolate reads homologous to RT for each species. Then, paralogous reads were pairwise compared using MEGA version 7 (Kumar et al., 2016) within each species and Kimura distances (Kimura, 1980) were calculated. Kimura distances were converted to times, expressed as millions of years ago (MYA), using a substitution rate of 1.3 × 10^−8^ defined in rice, as described by Ma and Bennetzen (2004).

### Genome Size Estimation

Root apices were collected from five *O. paniculata* plants and one rooted cutting of cv. Leccino, and fixed in ethanol:acetic acid (3:1 v/v). The apices were washed in an aqueous solution of 6 mM sodium citrate, 4 mM citric acid, treated with a mixture of 8% pectinase (Sigma), 2% macerozyme (Serva) and 7% cellulase (Calbiochem) in citrate buffer pH 4.6 for 45 min at 37°C, and then squashed under a coverslip in a drop of 60% acetic acid. The coverslips were removed after freezing at −80°C. The air-dried preparations (three slides for each *O. paniculata* plant and three for cv. Leccino) were simultaneously Feulgen stained after hydrolysis in 1 N HCl at 60°C for 8 min. After staining, the slides were subjected to three 10-min washes in SO_2_ water prior to dehydration and mounting in distyrene-dibutylphthalatexylene (DPX; BDH Chemicals). For each slide, 30 prophase nuclei were measured. Feulgen stained DNA in individual prophase nuclei was measured in images captured by a charge-coupled-device camera on a Leica DMRB microscope, using a Leica Q500MC image analyser. Results are given as average of 4C-DNA absorption value ± standard error (in arbitrary units).

### Fluorescence *in situ* Hybridisation

The *Copia-SIRE* probe, a 406 bp-long *Copia* fragment belonging to the *SIRE* lineage, was amplified by polymerase chain reaction (PCR) from both 50 ng of genomic DNA from *O. paniculata* and cv. Leccino. Primers were designed to an RNAse H encoding sequence (forward primer: 5′-TTGATCGAAAAAGCACTAG CGGAAC-3′ and reverse primer: 5′-AGTCCTCTACGAAT AAATGAAAAACG-3′) of a *SIRE*-related cluster from the graph-based clustering analysis. PCR conditions were 94°C for 4 min, followed by 30 cycles of 94°C for 30 s, 58°C for 30 s and 72°C for 40 s. A final extension was performed at 72°C for 7 min. PCR products were purified with a Wizard SV Gel and PCR Clean-Up System (Promega), and cloned into the pGEM-T Easy plasmid vector (Promega). The cloned fragments were sequenced. For each probe, one clone was selected (GenBank accession number OM829845 for *Copia-SIRE* probe of *O. paniculata* and OM829844 for *Copia-SIRE* probe of cv.Leccino) and used for FISH analysis.

Six olive probes designed on the sequences of TRs families specific for *O. europaea* were also used as: O-51 (905 bp, GenBank accession number OM829846), O-80 (879 bp, GenBank accession number OM829847), O-86 (889 bp, GenBank accession number OM829848), O-178 (1,025 bp, GenBank accession number OM829849), O-179 (1,145 bp, GenBank accession number OM829850) and O-218 (1,289 bp, GenBank accession number OM829851).

Primers used for O-51 were 5′-CCTATTGATGCT GTGTTGACC-3′ and 5′- GGATAGACTTTGTCCCGTGA-3′, for O-80 were 5′-GAAAAATGACGAAATTGCCCCCGA-3′ and 5′-TCGACTGTGTCGGAATTGGCTGAAATTTG-3′, for O-86 were 5′-TTTTTTCGTTTTTGGCGAATTGCT-3′ and 5′-CAGG GTTTTCCCAGTCACGACGT-3′, for O-178 were 5’-CGAA GAAGATTTGAGTTCAATCCA-3′ and 5’-GAAGAATGAGCAC TTTATATTTAGA-3′, for O-179 were 5′-ATAGAGAATAAGC AAAAGTCTACC-3′ and 5′-TGATGGTTTTAATATTGGAG CTT-3′ and for O-218 were 5’-CATTCCGACACCGATAAGAC-3′ and 5′-GGCCGAAATTTTGTAAGTTGT-3′. PCR conditions and cloning procedure were as described above.

Probes were labelled by nick translation using DIG-Nick Translation Mix (Roche) or Biotin-Nick Translation Mix (Roche).

*In situ* hybridisation was performed as described in Ceccarelli et al. (2010). Slides were prepared using root apices from potted plants for *O. paniculata*, or from cuttings for both cv. Leccino and *O. europaea* subsp. *cuspidata*. The apices were treated with a saturated aqueous solution of alpha-bromonaphtalene for 4 h at room temperature, fixed in ethanol:acetic acid (3:1 v/v) and processed as described above (see Genome Size Estimation). DNA of nuclei was denatured in a thermal cycler for 8 min at 70°C and the preparations were then incubated overnight at 37°C with 2 ng/μl of heat-denatured DNA probes. The digoxigenin and biotin at the hybridisation sites were detected by using sheep anti-digoxigenin-fluorescein (Roche) and streptavidin-Cy-3 (Sigma), respectively. Nuclei were then counterstained using 0.2 μg/ml 4,6-diamino-2-phenylindole (DAPI) in McIlvaine buffer pH 7.0, mounted in AF1 antifade solution (Citifluor) and examined with a Leica DMRB fluorescence microscope. At least ten metaphase plates were analysed for each probe and images were captured using an ILCE-7 camera (SONY) and optimised using Adobe Photoshop 5.0.

## Results

### Characterisation of the Repetitive Component in the Genus *Olea*

Genome structure of the genus *Olea* was studied in four taxa of the subgenus *Olea*, i.e. the cultivated olive (*O. europaea* subsp. *europaea*, cv. Leccino); *O. europaea* subsp. *cuspidata; O. europaea* subsp. *guanchica; O. exasperata*; and in *O. paniculata*, belonging to the subgenus *Paniculatae* (Table 1).

In order to identify different families of repeats, resulting samples of 100 nt paired-end reads were analysed with the RepeatExplorer2 tool. On average, repetitive DNA in *Olea* species ranged from 56% in *O. europaea* subsp*. guanchica* to 73% in *O. europaea* subsp. *cuspidata*, showing remarkable differences in terms of composition (Table 2). Our analysis indicated that the peculiar structure of the olive genome with the characteristic abundance of TR sequences (Barghini et al., 2014) was also present in other *Olea* taxa. In fact, the analysed genomes showed a massive occurrence of DNA satellites in the form of TRs, accounting from 23% in *O. europaea.* Subsp. *guanchica* to 50% in *O. europaea* subsp. *cuspidata*, with the notable exception of *O. paniculata*, for which TRs only amounted to 1.94% of the genome. For interspersed repeats, DNA TEs were poorly represented among the analysed taxa, while REs accounted for a considerable part of the repetitive component, ranging from 18.28% in *O. europaea* subsp. *cuspidata* to 51.59% in *O. paniculata.*

**Table 2:** Genome proportion of repetitive sequence classes among the analysed taxa.

Repeats in the genome	*O. europaea* subsp. *europaea*	*O. europaea* subsp. *guanchica*	*O. europaea* subsp. *cuspidata*	*O. exasperata*	*O. paniculata*
DNA-TE%	2.06	2.41	1.64	2.74	2.59
RE%	28.84	27.89	18.28	32.85	51.59
TR%	23.89	23.35	50.44	26.43	1.94
rDNA%	0.37	0.72	1.45	1.24	0.50
Not classified%	1.51	1.64	1.24	1.53	2.62
TOTAL%	56.67	56.01	73.04	64.80	59.25

### Analysis of Tandem Repeats

**Figure 1:**
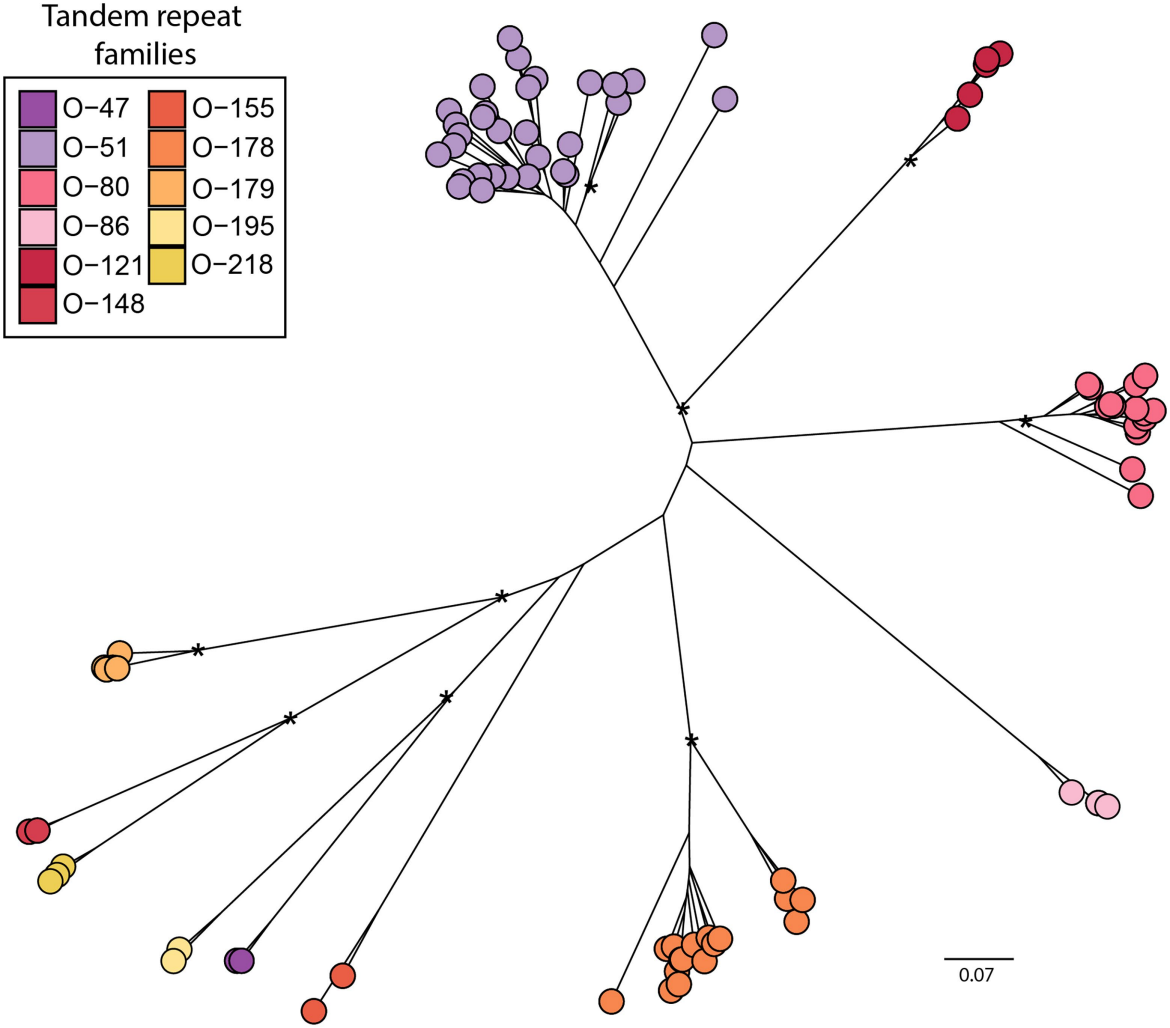
Distance tree of 11 TR families identified across the genus *Olea* (91 representative sequences). Bootstrap values higher than 0.6 are shown. Bar shows the nucleotide distance.

Clusters of *Olea* sequenced reads classified as putative satellites were inspected manually, in order to validate monomer consensus sequences. Overall, we identified 91 different sequences of TRs, organised in 11 major families (Figure 1 and Supplementary Figure S1). Among these major families, six had previously been identified in cultivated olive (Katsiotis et al., 1998; Bitonti et al., 1999; Minelli et al., 2000; Lorite et al., 2001; Barghini et al., 2014), even if their homologues were not found in all species by clustering analysis. In addition, five new species-specific families, three in *O. exasperata* and two in *O. paniculata* were identified by graph-based cluster analysis (Supplementary Figure S2). As already reported for cultivated olive, besides TR families with a typical monomer length of more than a hundred base pairs, some families were detected with repeat units of either 51-bp or 47-bp. TRs O-80, O-178 and O-218 constituted heavy satellite families, having a GC content around 44% or higher. By contrast, O-47, O-121 and O-51 had a GC content around 22, 27 and 32%, respectively, representing light satellite families (Supplementary Table S1).

**Figure 2:**
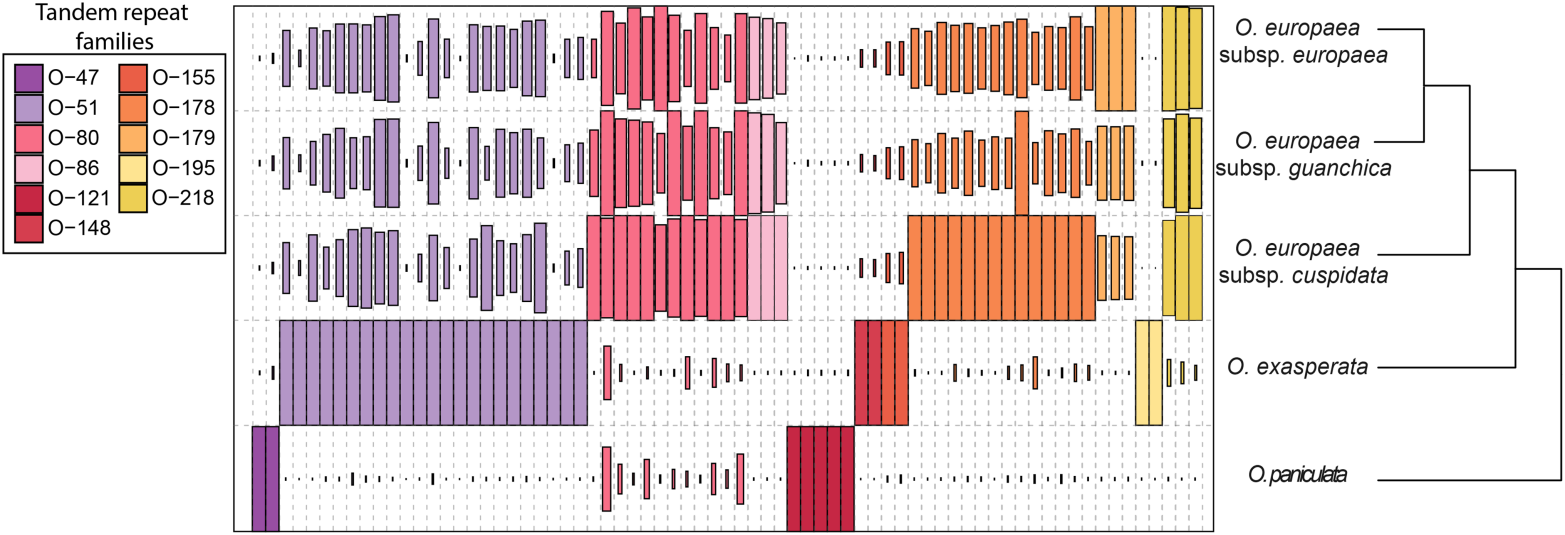
Sequence composition of TR sequences isolated from the analysed species. The size of the rectangle is proportional to the genome proportion of a cluster for each species. The colours of the rectangles correspond to the different TR families.

TR families showed great variability in terms of abundance across the genus *Ole*a. Mapping results indicated the presence of all sequences in all analysed taxa, highlighting a great genomic variability since some families were barely represented in one species while being highly abundant in another (Supplementary Table S1; Figure 2). Abundance data concerning TR families were also used to produce a phylogenetic tree (Figure 2). The dendrogram is consistent with the phylogeny of the genus *Olea* (Besnard et al., 2009), supporting separation among the three different sections analysed, with O. *paniculata*, the species closest to the *Olea* common ancestor, showing a TR abundance pattern quite different from the other species.

### Analysis of LTR-Retrotransposons

Besides TRs, LTR-RE-related clusters composed the bulk of highly and moderately repeated sequences in *Olea* genomes. After annotation against a library of 254 putative full-length REs of olive (Barghini et al., 2014), these elements were studied at the lineage level (Table 3). Seven lineages (plus one group that could not be annotated) were identified among *Copia* retrotransposons (*AleI-Retrofit*, *AleII*, *Angela*, *Bianca*, *Ivana-Oryco*, *SIRE* and *TAR/Tork*), and three lineages (plus one group that could not be annotated) were identified among *Gypsy* elements (*Athila*, *Chromovirus* and *Ogre/Tat*).

**Table 3:** Genome proportion of LTR-RE sequences and maximum percentage of variation among the analysed taxa.

Superfamily	Lineage	Genomic abundance					Max. percentage of variation
		*O. europaea* subsp. *europaea*	*O. europaea* subsp. *guanchica*	*O. europaea* subsp. *cuspidata*	*O. exasperata*	*O. paniculata*	
*Copia*	*AleI-Retrofit*	0.007	0.014	<0.005	<0.005	<0.005	68.65
	*AleII*	0.41	0.36	0.22	0.29	1.09	79.79
	*Angela*	3.55	3.48	2.23	1.93	5.28	63.36
	*Bianca*	0.67	0.74	0.38	0.59	0.60	47.73
	*Ivana-Oryco*	0.27	0.24	0.11	0.27	0.34	68.88
	*Maximus/SIRE*	1.07	1.03	0.64	0.49	6.13	92.00
	*TAR/Tork*	5.12	4.54	3.05	4.26	6.60	53.73
	Unknown	0.37	0.38	0.24	0.22	0.50	52.50
	*Total*	11.46	10.76	6.88	8.05	20.54	66.52
*Gypsy*	*Athila*	3.34	3.09	2.02	5.46	7.07	71.43
	*Chromovirus*	5.27	4.88	3.00	4.98	10.11	70.34
	*Ogre/Tat*	4.96	4.75	4.10	10.48	7.62	60.89
	Unknown	1.33	1.62	0.95	1.74	1.27	45.42
	*Total*	14.89	14.34	10.06	22.65	26.07	61.39
LTR-RE unclassified		0.38	0.35	0.20	0.27	2.18	91.03
LTR-*Gypsy*/LTR-*Copia*		1.30	1.33	1.46	2.81	1.27	54.88

Abundance of *Gypsy* LTR-REs ranged from 10.06% in *O. europaea* subsp. *cuspidata* to 26.07% in *O. paniculata*, and they were overrepresented compared to *Copia* elements, which ranged from 6.88% in *O. europaea* subsp. *cuspidata* to 20.54% in O*. paniculata*. The ratios of the genomic proportions of *Gypsy* and *Copia* elements differed among species, from 1.27 in *O. paniculata* to 2.81 in *O. exasperata*. Clusters that remained un-annotated composed a very small fraction of the analysed genomes, ranging from 0.20% in *O. exasperata* to 2.18% in *O. paniculata*.

**Figure 3:**
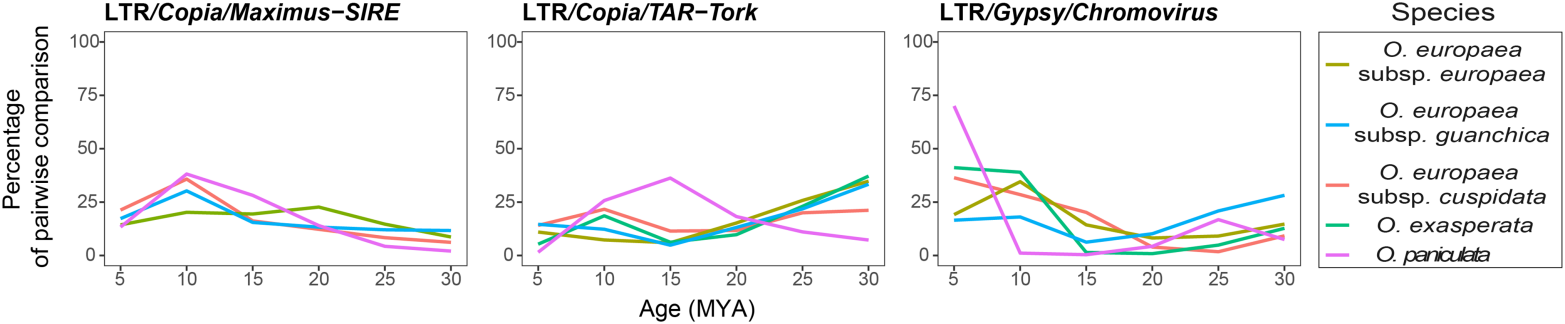
Timing of the LTR/*Copia/Maximus-SIRE*, *TAR-Tork* and LTR/*Gypsy/Chromovirus* retrotranspositional activity in the analysed taxa. The y-axis shows the percentage number of pairwise comparisons of reads matching the RE-RT-specific domain.

Furthermore, to elucidate the possible role of LTR-RE dynamics during *Olea* taxa separation, we also analysed RE insertion time (Figure 3). Although RE insertion times, calculated by comparing coding sequences (Ammiraju et al., 2007), should be taken cautiously, the results showed a similar proliferation profile for all the analysed taxa, except for *O. paniculata*, in which the proliferation burst of three major families of REs started in the last 25/20 million years (MY) and reached its apex in the last 15/5 MY.

### Repeats Composition Variation in *Olea* Taxa

**Figure 4:**
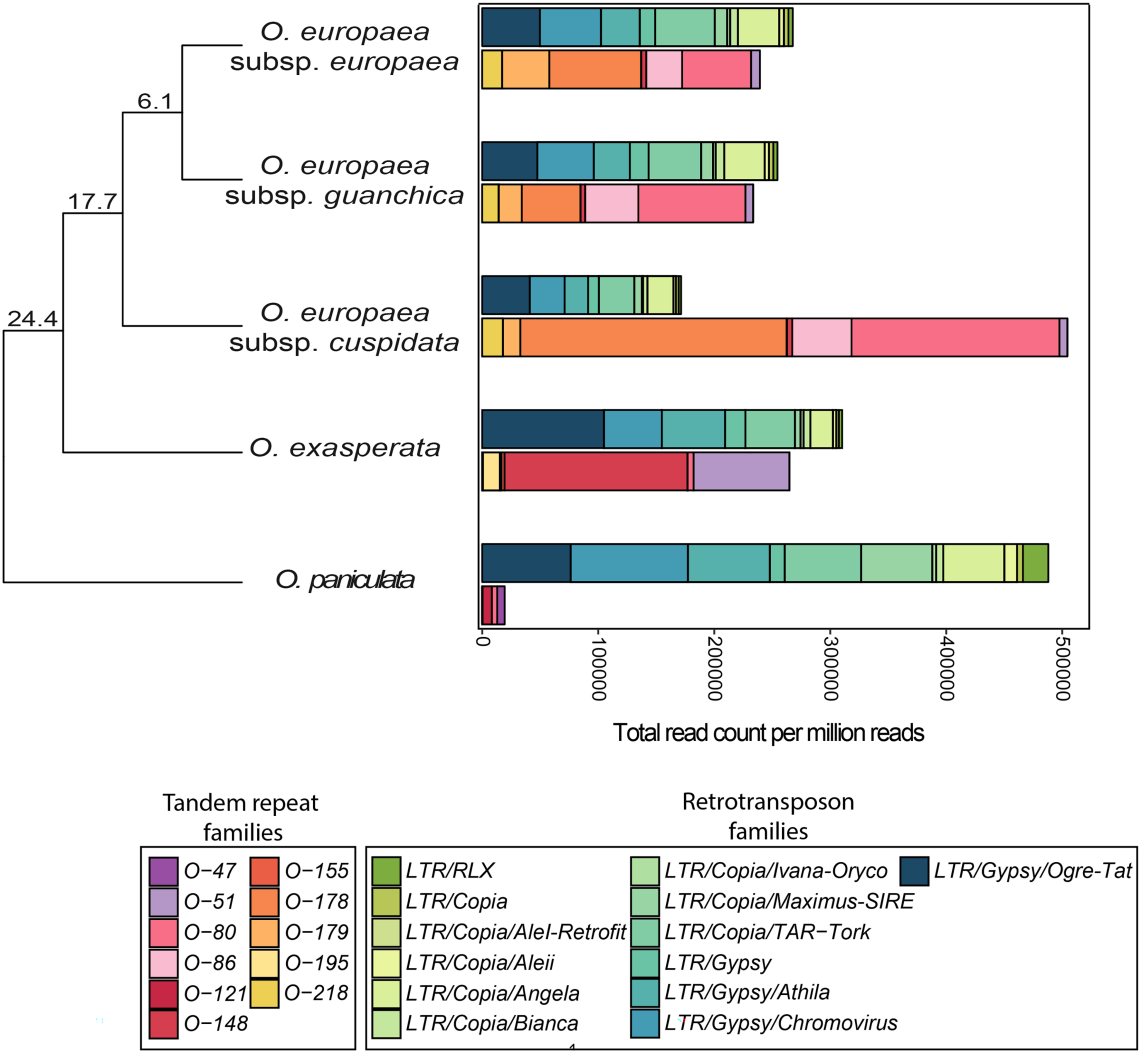
Stacked bar plots comparing the genome proportion of LTR-RE families and TR families in *Olea*. Abundance values were measured by counting the number of reads (per million) mapping the set of repetitive sequences collected in the reference library. Phylogenetic tree reports the estimated divergence times (in MY) from the common ancestor for the *Olea* taxa used in this study, according to Besnard et al. (2009).

Comparing the abundance of RE and TR families retrieved in the 5 taxa analysed, it can be seen that in four of them TR abundance was inversely correlated with that of REs (Figure 4). The opposing trend was observed for *O. paniculata*, potentially the oldest species, originated around 24 million years ago (MYA) from the *Olea* common ancestor (Besnard et al., 2009), which had very few tandem-repeated sequences, while being rich in LTR-REs.

### Cytological Analyses

The differences in repeat organisation between *O. paniculata* and the other taxa were confirmed by cytological analyses. Image cytometry of prophase nuclei was used to estimate the genome size of *O. europaea* subsp. *europaea* and *O. paniculate.* The analyses returned a 4C-DNA absorption value of 207,067 ± 5,673 for *O. europaea* subsp. *europaea* and 376,475 ± 46,638 for *O. paniculate*, respectively, indicating that *O. paniculata* genome size was larger than that of *O. europaea* subsp. *europaea*, showing an increase of 44.9%.

**Figure 5:**
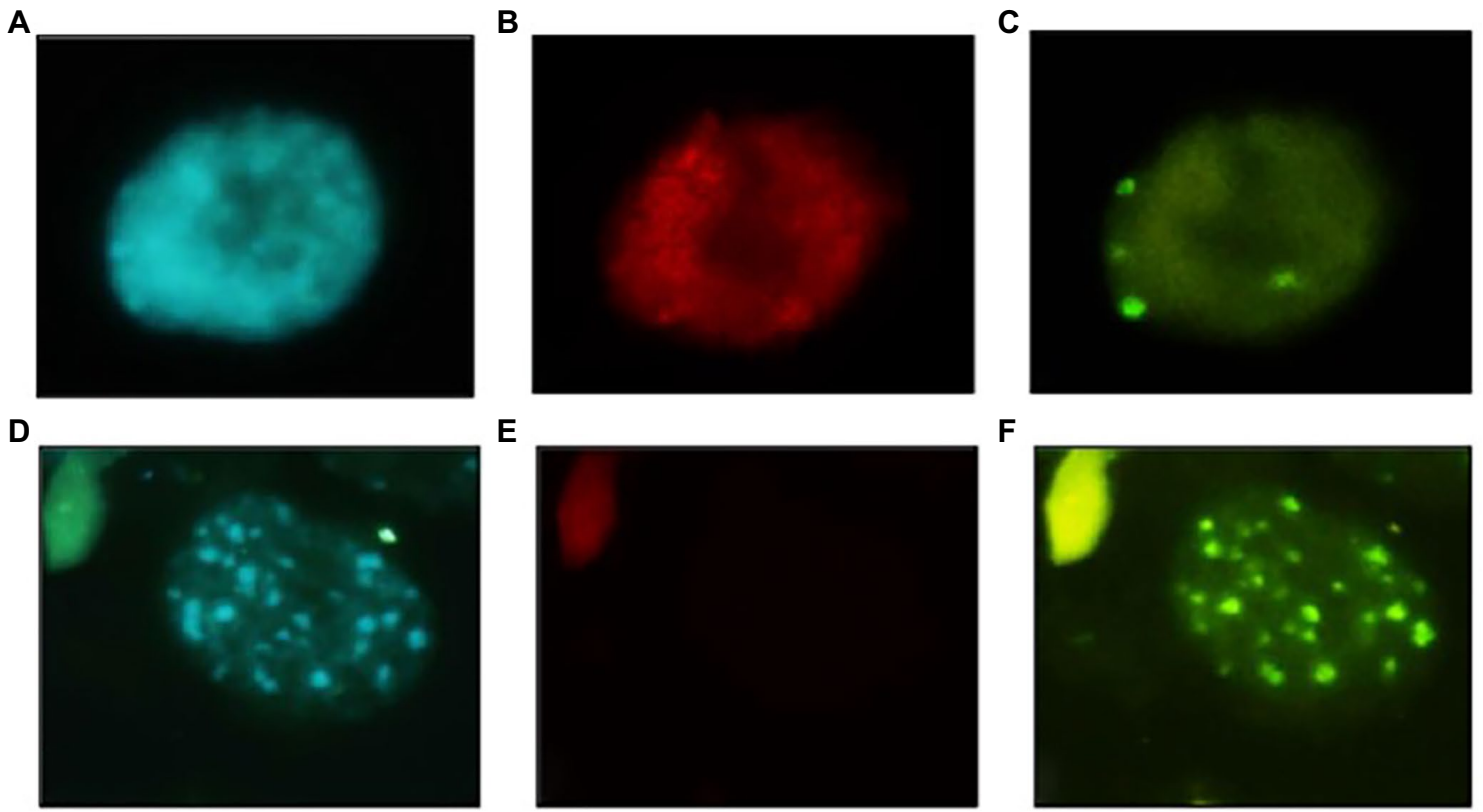
Interphase nuclei in the shoot meristem of *Olea paniculata* **(A**–**C)** and *Olea europaea* subsp. *europaea* **(D**–**F)**. Images after DAPI staining **(A,D)**, after hybridisation with the *O. paniculata Copia-SIRE* probe **(B,E)** and after hybridisation with *Oe*Taq80 DNA repeats **(C,F)**. Images similar to **(B,E)** were obtained with the *O. europaea* subsp. *europaea Copia-SIRE* probe (data not shown). Bar = 10 μm.

The variation in genome size was reflected in the chromatin organisation. Indeed, *O. paniculata* interphase nucleus, largely occupied by LTR-REs, showed an eureticulate structure, characterised by dense, conspicuous and regular chromatin reticulum with barely visible chromocenters (DAPI positive heterochromatic regions; Figure 5A), while cultivated olive had an areticulate or chromocentric nucleus, with prominent chromocenters standing out on a barely visible euchromatin reticulum (Figure 5D). Fluorescence *in situ* hybridisation (FISH) of a fragment belonging to a family of *Copia-SIRE* LTR-REs confirmed their massive presence in *O. paniculata*, being the hybridisation signal largely scattered across the nucleus (Figure 5B). By contrast, the signal from hybridisation of a TR fragment from the family O-80 (*Oe*Taq80) formed a few small clusters corresponding to as many chromocenters (Figure 5C). The opposite results were obtained in the nuclei of cultivated olive, where no signal was observed after FISH with the *Copia*-SIRE probe (Figure 5E), but intense hybridisation signals of *Oe*Taq80 were localised at the DAPI positive chromocenters (Figure 5F).

**Figure 6:**
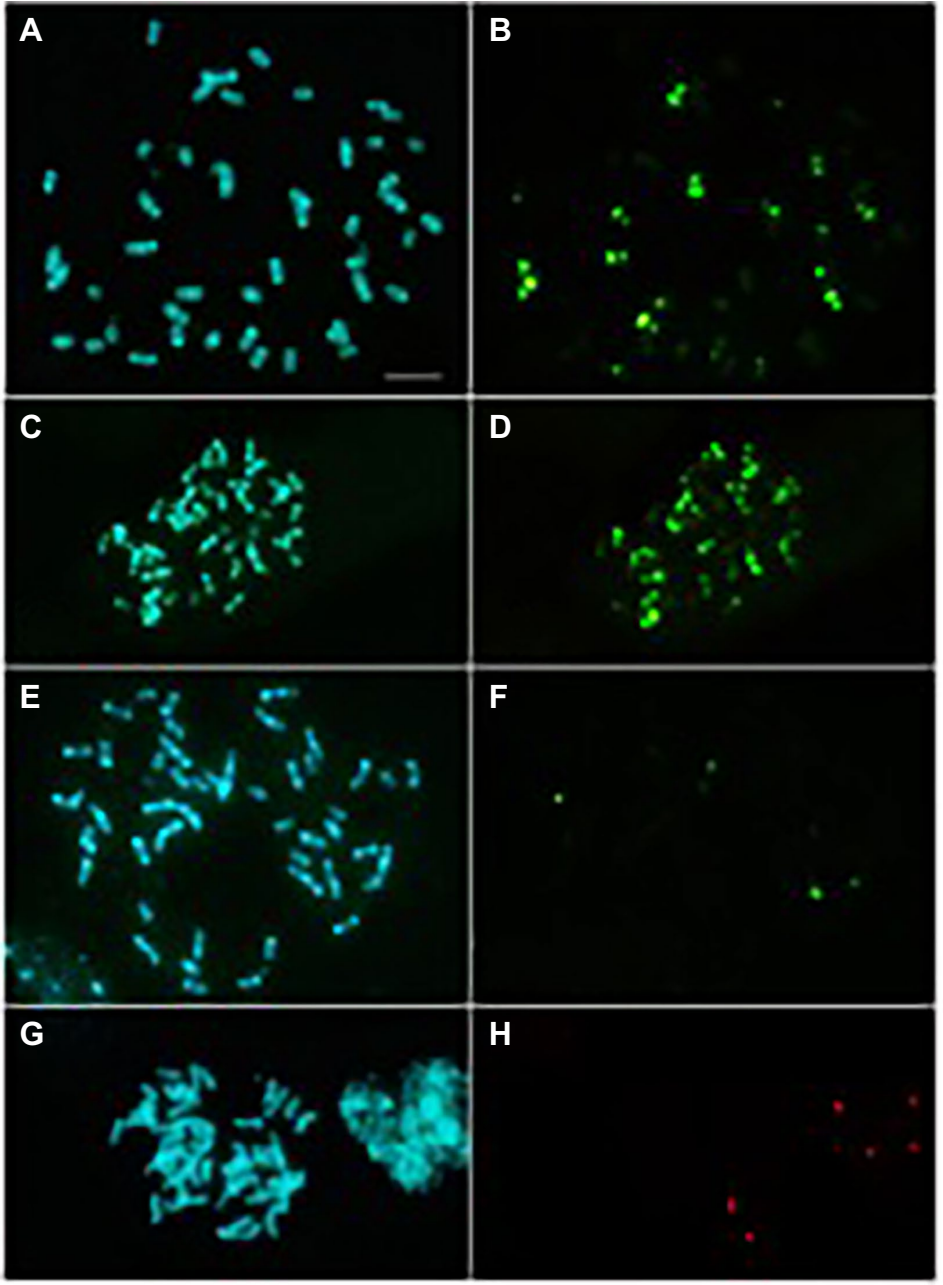
Metaphase plates of *O. europaea* subsp. *europaea* [cv. Leccino; **(A,B,E,F)** and *O. europaea* subsp. *cuspidata* **(C,D,G,H)** after DAPI staining **(A,C,E,G)** and hybridisation with O-178 (**B,D**; fluorescein) or O-51 (**F,H**; fluorescein) repeats. Bar = 10 μm.

Finally, FISH experiments were carried out to highlight possible differences in TRs chromosomal localization between cultivated olive and *O. europaea* subsp. *cuspidata*, for which molecular analyses indicated a TR abundance of 50% of the genome. Six different probes were designed on the sequences of TRs families specific for *O. europaea* and hybridised in root-tips chromosomes of the two subspecies. O-51 and O-179 families had never been hybridised before, whereas the chromosomal localization of the remaining TRs was already studied by Katsiotis et al. (1998) and Minelli et al. (2000) in different olive cultivars. Metaphase plates hybridised with O-51 and O-178 were reported in Figure 6; those hybridised with O-80, O-86, O-179 and O-218 were reported in Supplementary Figure S3.

The maximum number of chromosome pairs showing signals after hybridisation with each probe, and minimum and maximum number of hybridisation signals counted on metaphase plates in the two subspecies were reported in Table 4. Differences in chromosomal distribution of O-178 and O-51 related sequences were found between the two taxa. Ten chromosome pairs of the cultivated olive complement showed O-178 hybridisation signals versus the 15 chromosome pairs in *O. europaea* subsp. *cuspidata*. In total, 47 to 50 hybridisation signals were counted on *O. europaea* subsp. *cuspidata* chromosomes while only 22 to 30 signals were found in cultivated olive (Table 4). On the contrary, O-51 probe found nucleotide sequence homology in two chromosome pairs of the cv. Leccino complement and only in one pair in *O. europaea* subsp. *cuspidata* (Table 4).

**Table 4:** Maximum number of chromosome pairs showing signals after hybridisation with each probe, and minimum and maximum number of hybridisation signals counted on metaphase plates in the two subspecies.

Probe	Chromosomes pairs		Hybridisation signals	
	*O. europaea* subsp*. europaea*	*O. europaea* subsp. *cuspidata*	*O. europaea* subsp. *europaea*	*O. europaea* subsp. *cuspidata*
O-51	2	1	3–4	2
O-80	23	23	63–66	54–62
O-86	13	13	29–37	27–35
O-178	10	15	22–30	47–50
O-179	17	17	37–40	40–42
O-218	10	10	18–20	15–19

At least ten metaphase plates for each probe and subspecies were analysed.

Any noticeable difference was found between the two subspecies regarding the chromosomal distribution of the other TRs (Table 4; Supplementary Figure S3). O-80-related sequences were found in all the chromosome pairs in both taxa. Structural heterozygosity of the chromosome pair I, already described in cultivated olive (cv. Coratina; Minelli et al., 2000), was also observed in both cv. Leccino and subsp. *cuspidata.* O-86 repeats hybridised on 13 chromosome pairs. The O-179 probe found related sequences in 17 pairs of both chromosome complements. A slightly higher number of weak hybridisation signals related to O-218 sequences was observed in cv. Leccino, the two complements substantially showing the same number of signals of major and minor intensity (Table 4).

## Discussion

Repetitive sequences represent one of the most cryptic components of eukaryotic genomes (Garrido-Ramos, 2015, 2017; Bourque et al., 2018). For a long time, this fraction was considered of little importance, and it still remains ill-defined because of the technical issues associated with reliable characterising representative sets of sequence and also for the great variability in terms of abundance and/or sequence conservation at interspecific and intraspecific levels (Mascagni et al., 2015, 2017a; Robledillo et al., 2018).

In order to clarify the processes that led to the present structure of the cultivated olive genome, a deep characterisation of the repetitive fraction of olive was performed in comparison with four other taxa belonging to the genus *Olea*, through bioinformatics, cytophotometric and cytological analyses. To achieve this, first, a graph-based clustering approach, already applied in several species (Novák et al., 2014; Barghini et al., 2015; Usai et al., 2017), including cultivated olive (Barghini et al., 2014a), was used. Results confirmed the peculiar genomic structure of cultivated olive, with its high composition of TRs (accounting for ~24%). The high abundance of TRs was also shown to be a general feature of all the analysed species of the subgenus *Olea*, with *O. europaea* subsp. *cuspidata* having a TR abundance of 50% of the genome. These data confirmed the singular evolution of the subgenus *Olea* since, in other taxa, TRs usually account for <10% of the genome, with some exceptions like cucumber or *Fritillaria falcata*, whose genomes comprise ∼23 and 36% of these sequences, respectively (Huang et al., 2009; Ambrožová et al., 2010).

The TR families identified in the analysed genomes showed low sequence similarity and great variability in terms of genomic abundance, suggesting their independent origins. In plants, it is a common feature of related species to share a set of TR families, with one or a few predominant TR species-specific families (King et al., 1995). However, TR sequences are usually considered fast-evolving components that can also cause reproductive barriers between organisms, thus promoting species separation (Schmidt and Heslop-Harrison, 1993; Garrido-Ramos, 2017). In fact, while some TR sequences can exhibit conservation of the monomer sequence for long evolutionary periods (Cafasso and Chinali, 2014; Mehrotra and Goyal, 2014), other TRs are subjected to different constraints. Low preservation of sequence similarity or abundance is reported for several plant groups, where some monomers may be preferred over others at the evolutionary level (Flavell, 1982; Cafasso and Chinali, 2014; Mehrotra and Goyal, 2014). Recently, the hypothesis of a possible contribution to TR evolution and mobility by TEs has been proposed (Meštrović et al., 2015; Vondrak et al., 2020). In the genomes of *Chenopodium sensu stricto*, TEs may act as a substrate for TRs, generating a sort of ‘library’ of tandemly arranged sequences that, after being dispersed through the genome through transposition, may be amplified into long arrays of new TR families (Belyayev et al., 2020).

Since relative abundance of well-represented repeats is a representation of general genome composition, we used genome-wide abundance of TRs as continuously varying characters in order to build a phylogenetic tree. This methodology can be particularly useful in groups showing little genetic differentiation in classic phylogenetic markers, actually providing information for phylogenetic inference (Dodsworth et al., 2014). The dendrogram obtained from our data supported the separation among the three sections of *Olea* considered in this study (Besnard et al., 2009), highlighting the differences in the genome composition of O. *paniculata*, the closest species to the *Olea* common ancestor.

In *O. paniculata*, as typical of many plant species, interspersed REs accounted for the vast majority of the repetitive component, while TRs were barely present, consistently with the results reported for a TR family by Bitonti et al. (1999). In this species, our data indicated that massive RE proliferation started around ~20 MYA and reached its apex in the last 15–5 MY, i.e., after separation of the subgenus *Olea*. Concurrently, the other *Olea* species originating from the same ancestor (Besnard et al., 2009) had a huge increase in TR abundance which can be explained by the so-called ‘library model’ (Fry and Salser, 1977). In this hypothesis of TR evolution, closely related species share a set of conserved TR families each of which is differentially amplified in each species forming a sort of library accompanied by rapid evolution of nucleotide sequences and copy number change (Cesari et al., 2003; Thakur et al., 2021). In Olea, the partial replacement of an RE increase by TR accumulation, during subgenus *Olea* species separation, was a fairly unique event. Interestingly, in all species overall, TR abundance was inversely correlated to that of REs. This trend might imply a direct competition in the proliferation of these two classes of repeats, suggesting that the species of the subgenus *Olea* underwent amplification of TRs and a reduced proliferation of retrotransposons.

Cytological analyses underlined the differences in genome size and organisation of *O. paniculata* compared to *O. europaea* subsp. *europaea*. The genome size of *O. paniculata* was about 50% larger than that of cultivated olive. Such a difference between species with the same chromosome number is usually attributed to variations in the abundance of repetitive DNA (Flavell, 1986). In this case, supported by RE insertion timing data and by *in situ* hybridisation results, the genome expansion of *O. paniculata* might be derived from a massive amplification through retrotransposition of major individual RE families in the last ~20 MY, while TRs remained below 2% of the genome. A similar case is represented by a study on the genus *Passifora*, where *Passifora quadrangularis*, the species with the largest genome, presents a higher accumulation of REs compared to *Passifora organensis*, whose genome shows a greater diversity and the highest proportion of satellites (Sader et al., 2021).

Accordingly, there are reports of how the amplification of one or a few specific repeats led to an increase in genome size. In maize, almost 25% of the genome is represented by five LTR-RE families (SanMiguel et al., 1996). In five species of iris (*Iris* ser. *Hexagonae*), a characteristic RE type accounts for 6–10% of the genome (Kentner et al., 2003). Finally, in *Vicia pannonica*, a single family of *Gypsy* elements caused the expansion of the genome by 50% (Neumann et al., 2006).

The different composition of the *O. paniculata* genome also reflects in the organisation of its genetic material. Indeed, interphase nuclei are arranged in distinct reticulate structures (eureticulate type; Delay, 1946-1947, 1948) confirming the absence of highly repetitive TR families. In *O*. *europaea* subsp. *europaea*, the proliferation of TRs, which still represents an important part of its repetitive component, could have preserved the genome from massive expansion. Moreover, the great amount of TRs, which are the main component of heterochromatin, regulating its formation and preserving its structure (Grewal and Elgin, 2007; Garrido-Ramos, 2015), results in the occurrence of chromocenters, nuclear regions containing just highly repetitive, tandemly arranged DNA sequences (Botchan et al., 1971; Gall et al., 1971; Peacock et al., 1974; Guenatri et al., 2004). This phenomenon is not limited to plant kingdom: even in some animal genomes, it is possible to observe cases in which TEs likely affected the formation of TRs and the conversion of euchromatic chromosomes into heterochromatic ones (Bachtrog et al., 2019; Palacios-Gimenez et al., 2020).

Finally, FISH experiments highlight that some TRs were physically localised in the genome of closely related species (i.e., *O. europaea* subsp. *europaea* and subsp. *cuspidata*) significantly differing in TRs abundance. The results suggested a different evolutionary model for the various families within *O. europaea*. A higher number of hybridisation signals was observed for O-178 in *O. europaea* subsp. *cuspidata* rather than in subsp. *europaea*. In this case, it is clear that O-178 dissemination in a genome (involving TEs or other mechanisms) occurred more extensively than in the other one. On the contrary, O-51 showed 2 hybridisation signals in *O. europaea* subsp. *europaea* versus only one in *O. europaea* subsp. *cuspidata*. However, it is to be considered that O-51 accounted only for a minimal portion of the genomes. Concerning the other TRs, regardless of their genome abundance, a similar number of hybridisation signals were observed for O-80, O-86, O-179 or O-218 families in the two subspecies. It can be assumed that, after their dissemination throughout the *O. europaea* genome, these TR families differentially amplified in the two subspecies, maintaining the same positions in each genome. However, it cannot be ruled out that differences in genomic abundance not revealed by cytological observations could be due to the greater distribution in a genome of short arrays whose copy number is below the sensitivity FISH threshold (Ruiz-Ruano et al., 2016). In conclusion, the current study shed light on the evolution of the genus *Olea*, highlighting the prominent role of TRs in fostering genome structure variation. After the separation of the subgenus *Olea* (24.4 MYA), tandemly arranged sequences underwent a massive proliferation, leading to the peculiar genomes of cultivated olive and its related species. By contrast, in *O. paniculata*, the closest species to the *Olea* common ancestor, the TR proliferation burst never occurred, opening the way for REs amplification, which resulted in an expansion of the genome. Based on the huge difference in repetitive fraction composition, combined with the notable TR abundance of some species, the genus *Olea* represents a quite singular model of genome evolution in higher plants. Studies, using new long-molecule sequencing methods, will further decipher the structure of TR loci and help to clarify the amplification mechanisms of these sequences.

## Data Availability Statement

The datasets presented in this study can be found in online repositories. The names of the repository/repositories and accession number(s) can be found at: https://www.ncbi.nlm.nih.gov/, PRJNA810942, SRX465835 https://www.ncbi.nlm.nih.gov/genbank/, OM829845 https://www.ncbi.nlm.nih.gov/genbank/, OM829844 https://www.ncbi.nlm.nih.gov/genbank/, OM829846 https://www.ncbi.nlm.nih.gov/genbank/, OM829847 https://www.ncbi.nlm.nih.gov/genbank/, OM829848 https://www.ncbi.nlm.nih.gov/genbank/, OM829849 https://www.ncbi.nlm.nih.gov/genbank/, OM829850 https://www.ncbi.nlm.nih.gov/genbank/, OM829851.

## Author Contributions

FM, AC, and LN planned and designed the project. TG and LB performed nucleic acid extractions. MC and CT performed the cytological analyses. FM and EB performed the bioinformatics analyses. FM, EB, MC, LB, CT, CD, TG, LN, and AC discussed the data, wrote the manuscript, and contributed to its final form. All authors contributed to the article and approved the submitted version.

## Funding

This research was supported by the Department of Agriculture, Food and Environment of the University of Pisa, Italy, Project ‘Plantomics’.

## Conflict of Interest

CT was employed by CSIRO Agriculture & Food, Narrabri, NSW (Australia).

The remaining authors declare that the research was conducted in the absence of any commercial or financial relationships that could be construed as a potential conflict of interest.

## Publisher’s Note

All claims expressed in this article are solely those of the authors and do not necessarily represent those of their affiliated organizations, or those of the publisher, the editors and the reviewers. Any product that may be evaluated in this article, or claim that may be made by its manufacturer, is not guaranteed or endorsed by the publisher.
