# Supplementary material for: The Singular Evolution of Olea Genome Structure
Source: Front Plant Sci. 2022 Mar 31;13:869048. doi: 10.3389/fpls.2022.869048 (PMC9009077; doi:10.3389/fpls.2022.869048)
Supplement: Supplementary file 1 [file Table_1.DOCX]

|  |  | *O. europaea* subsp. *europaea* | | *O. europaea* subsp. *guanchica* | | *O. europaea* subsp. *cuspidata* | | *O. exasperata* | | *O. paniculata* | |
| --- | --- | --- | --- | --- | --- | --- | --- | --- | --- | --- | --- |
| TR | Monomer [bp] | Genomic abundance | G+C  [%] | Genomic abundance | G+C  [%] | Genomic abundance | G+C  [%] | Genomic abundance | G+C  [%] | Genomic abundance | G+C  [%] |
| O-47 | 47 | < 0,001 |  | 0,001 |  | < 0,001 |  | 0,001 |  | 0,646 | 22.30 |
| O-51  * | 51 | 0,756 | 33.60 | 0,663 | 32.90 | 0,674 | 33.43 | 8,234 | 31.95 | 0,003 |  |
| O-80  ** | 80 | 5,935 | 48.10 | 9,232 | 47.90 | 17,930 | 47.17 | 0,521 |  | 0,457 | 46.90 |
| O-86  *** | 86 | 3,082 | 35.45 | 4,577 | 36.00 | 5,107 | 35.45 | 0,004 |  | 0,002 |  |
| O-121 | 121 | < 0,001 |  | >0,001 |  | < 0,001 |  | 0,001 |  | 0,794 | 27.44 |
| O-148 | 148 | 0,424 |  | 0,402 |  | 0,464 |  | 15,744 | 47.52 | 0,002 |  |
| O-155 | 155 | 0,025 |  | 0,023 |  | 0,021 |  | 0,266 | 54.50 | 0,001 |  |
| O-178 **** | 178 | 7,891 | 44.28 | 5,039 | 44.10 | 22,953 | 44.65 | 0,131 |  | 0,026 |  |
| O-179 ***** | 179 | 4,058 | 37.70 | 1,990 | 36.60 | 1,503 | 38.50 | 0,004 |  | 0,004 |  |
| O-195 | 195 | < 0,001 |  | 0,002 |  | < 0,001 |  | 1,462 | 41.15 | < 0,001 |  |
| O-218  ****** | 218 | 1,720 | 45.40 | 1,424 | 45.00 | 1,785 |  | 0,062 |  | 0,001 |  |

Supplementary Table S1. Tandem repeat families investigated in this study.

*Already known as Oe51 (Barghini et al. 2014)

**Already known as OeTaq80 (Bitonti et al. 1999)

***Already known as OeGEM86 (Bitonti et al. 1999)

****Already known as 178 (Lorite et al. 2001)

*****Already known as Oe179 (Barghini et al. 2014)

******Already known as pOSE218 (Katsiotis et al. 1998)
