# Supplementary material for: The Singular Evolution of Olea Genome Structure
Source: Front Plant Sci. 2022 Mar 31;13:869048. doi: 10.3389/fpls.2022.869048 (PMC9009077; doi:10.3389/fpls.2022.869048)

Supporting Information

The Singular Evolution of Olea Genome Structure

Mascagni F.*,[1], Barghini E.[1], Ceccarelli M.[2], Baldoni L.[3], Trapero C.[4], Díez C. M.[5], Natali L.[1], Cavallini A.[1], Giordani T.[1]

*Corresponding author: E-mail: flavia.mascagni@unipi.it*Corresponding author: flavia.mascagni@unipi.it

Supplemental Figure S1. Sequence LOGOs of the 11 families of identified tandem repeats

O-47

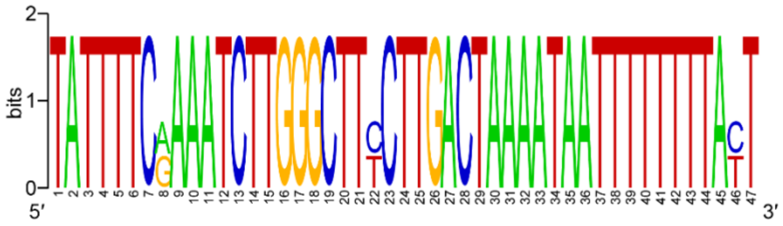

O-51

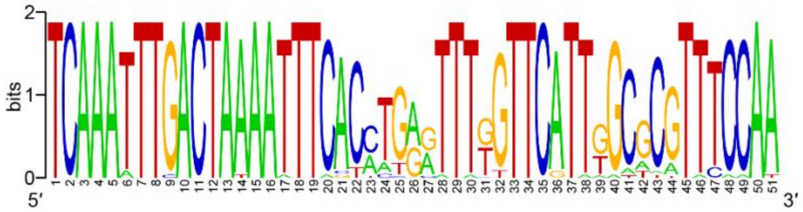

O-80

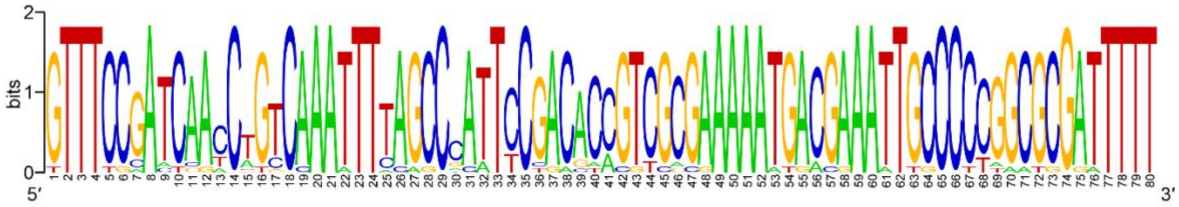

O-86

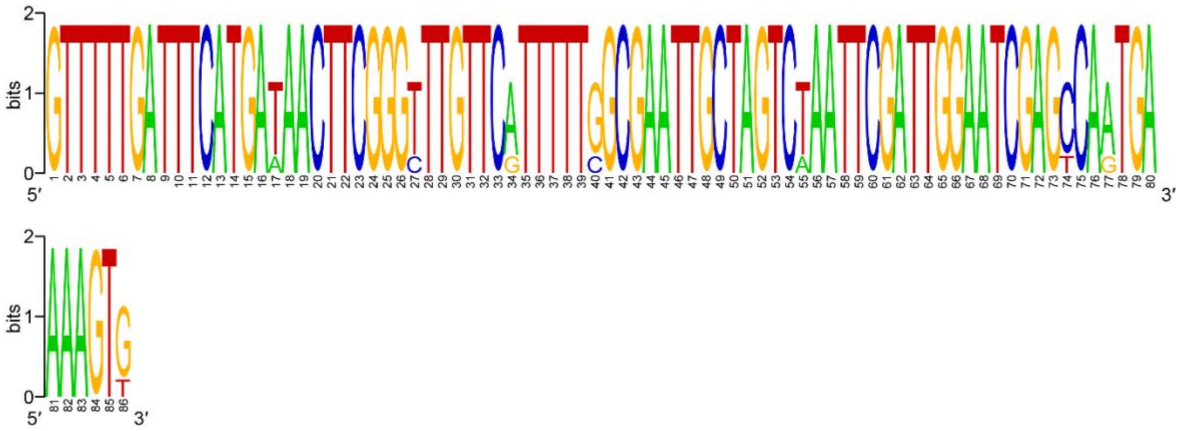

O-121

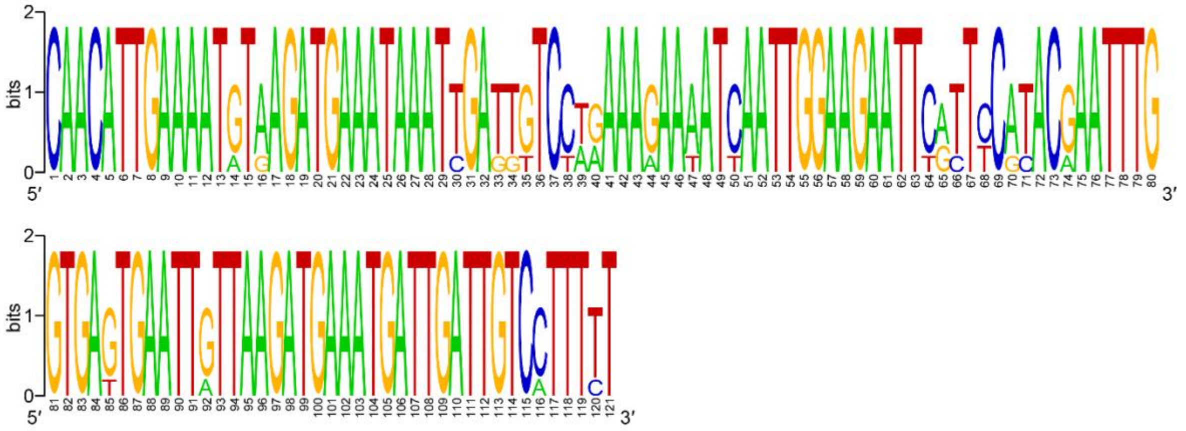

O-148

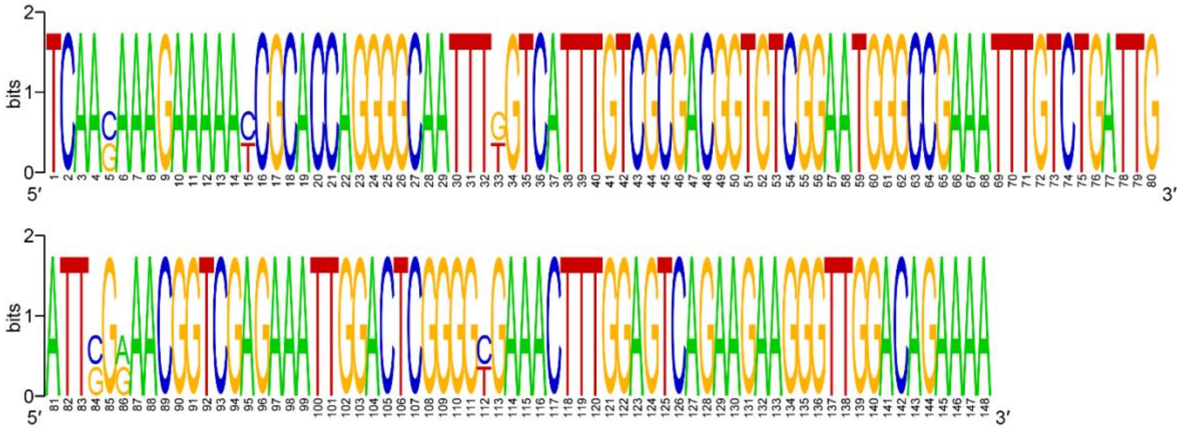

O-155

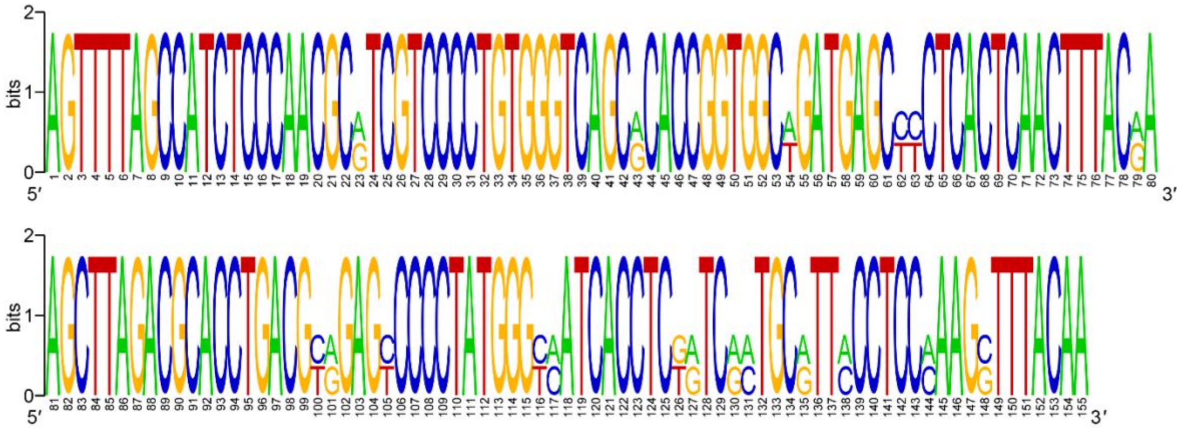

O-178

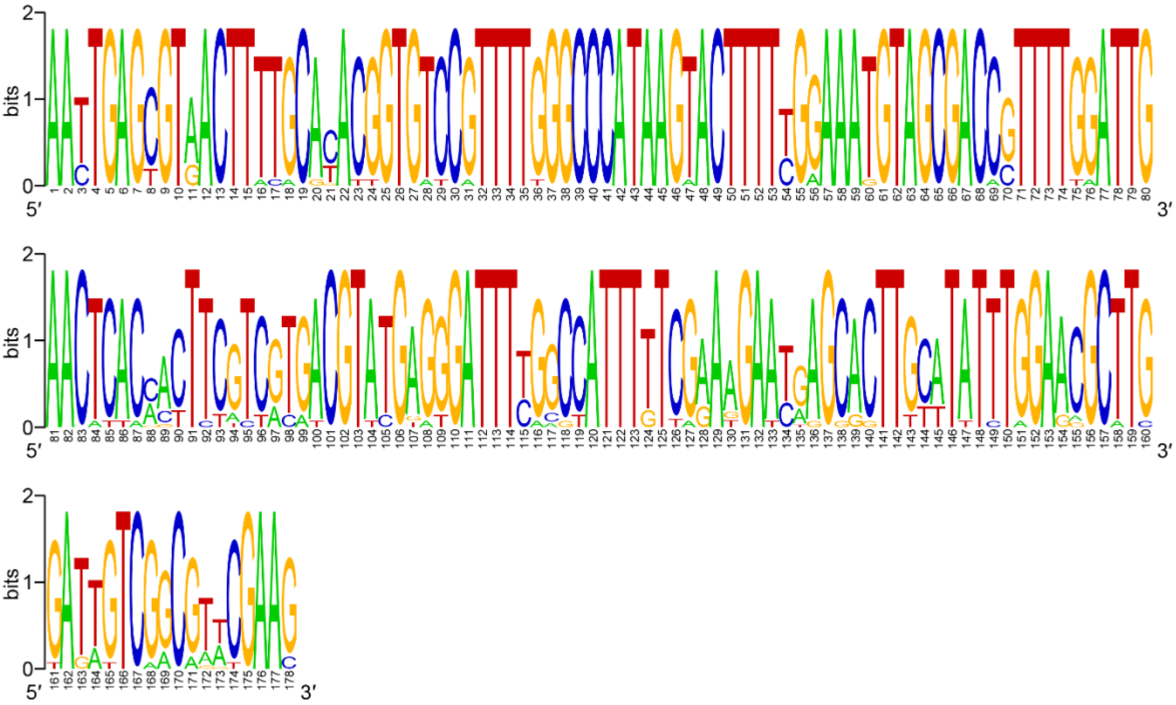

O-179

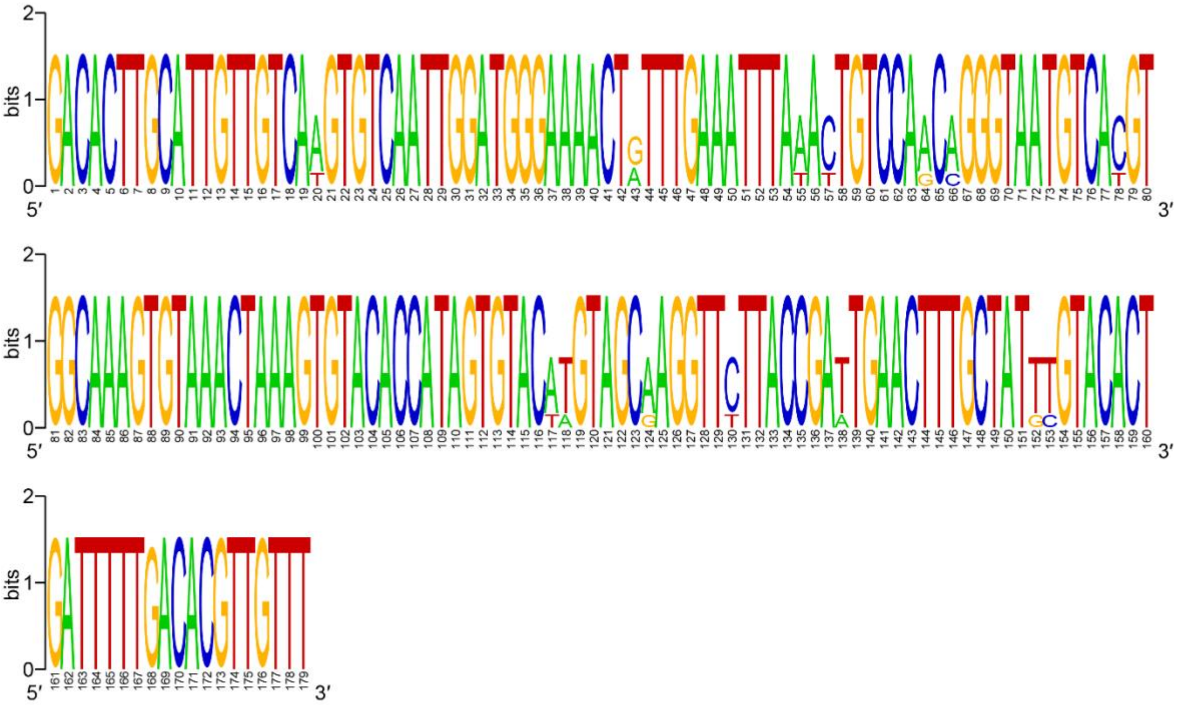

O-195

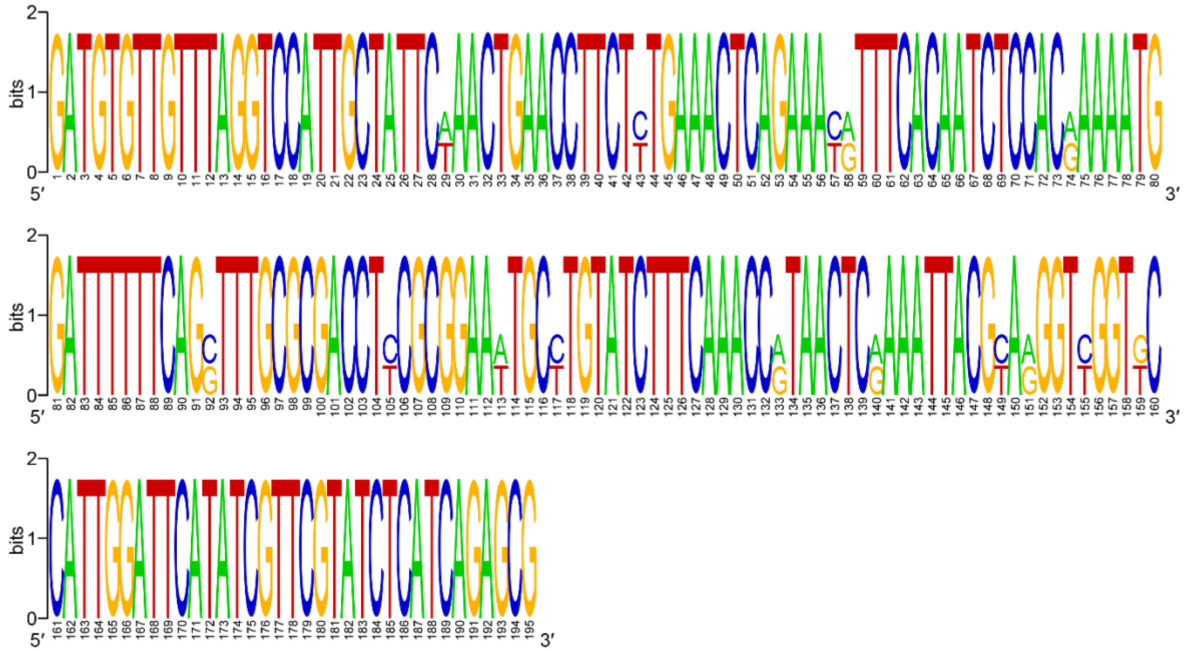

O-218

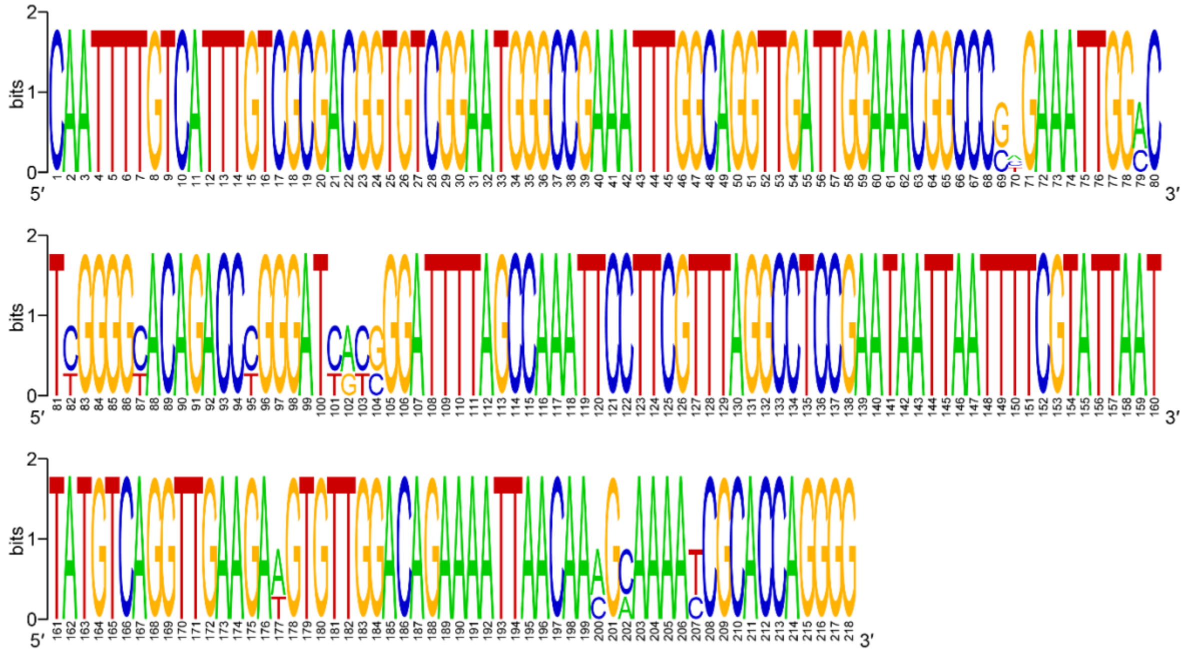

Supplemental Figure S2. Graph representation of the 5 newly discovered family of tandem repeat

O-47

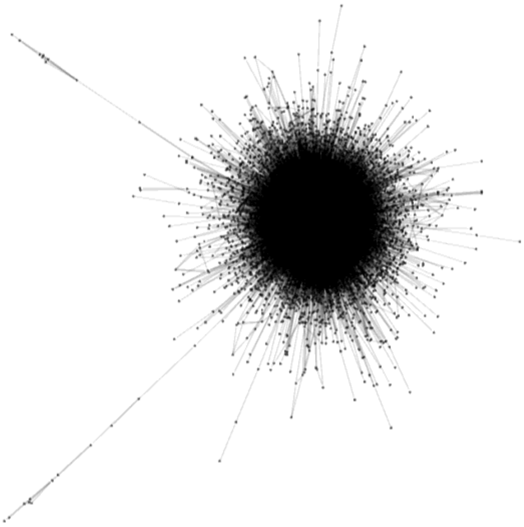

O-121

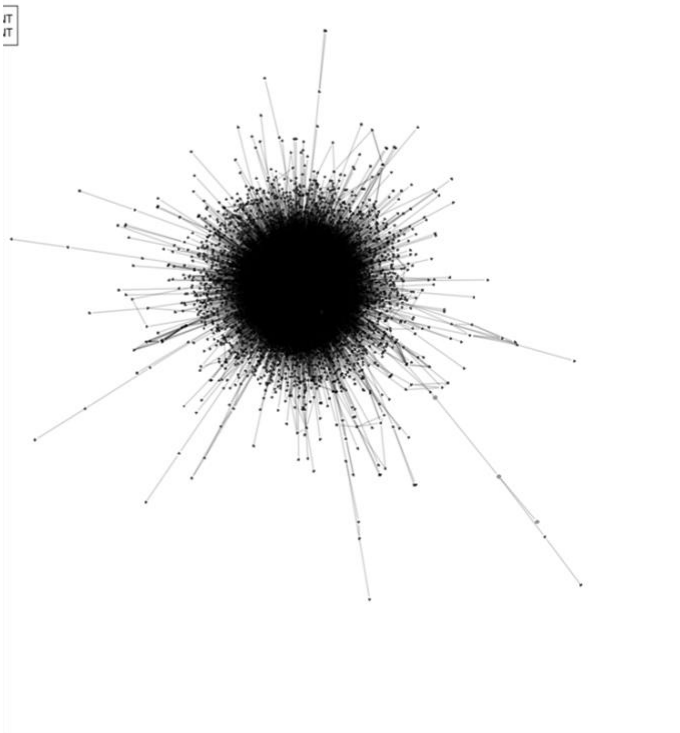

O-148

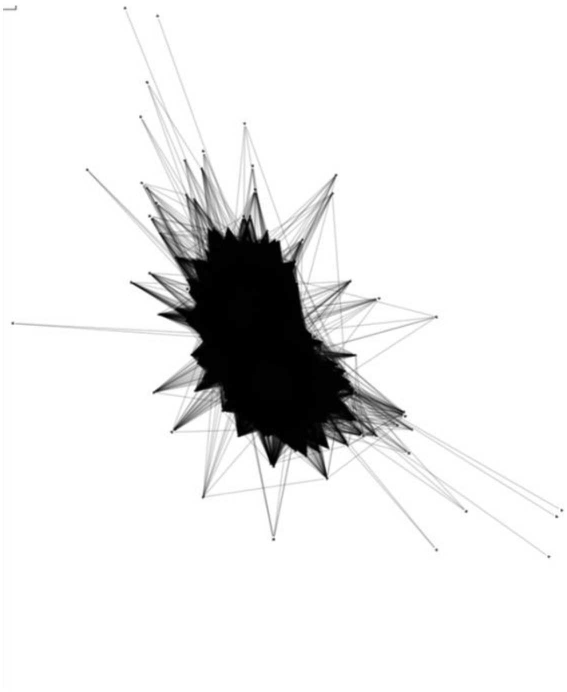

O-155

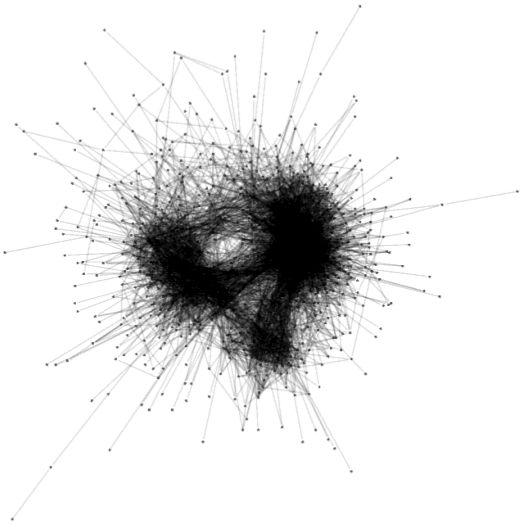

O-195

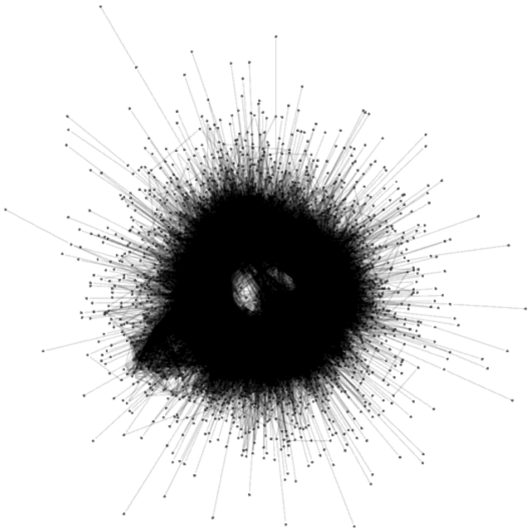

Supplemental Figure S3. Metaphase plates of *O. europaea* subsp. *europaea* (cv. Leccino; A, B, E, F, I, J, M, N) and *O. europaea* subsp. *cuspidata* (C, D, G, H, K, L, O, P) after DAPI staining (A, C, E, G, I, K, M ,O) and hybridization with O-80 (B and D; fluorescein), O-869 (F and H; Cy3), O-179 (J and L; fluorescein), or O-218 (N and P; Cy3) repeats. Bar = 10 µm.

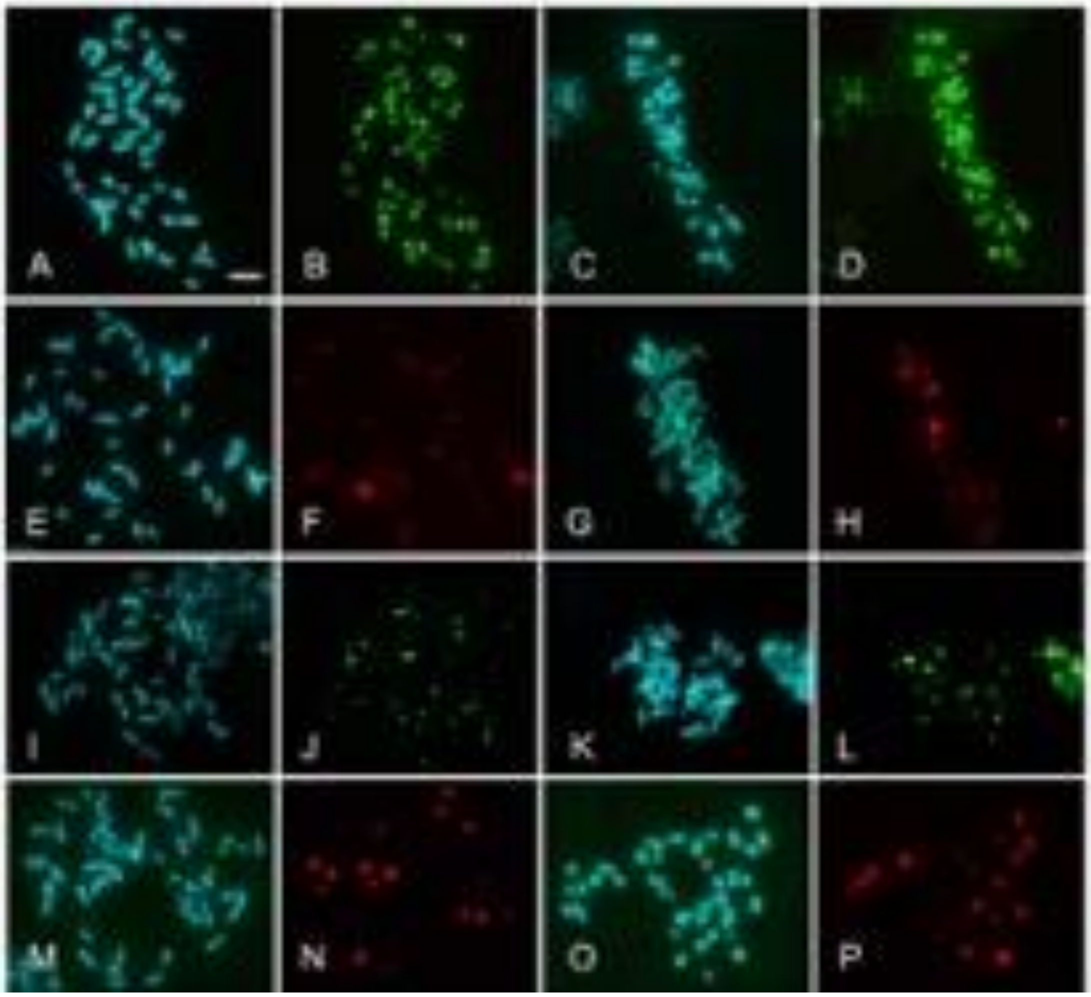

Supplement: Supplementary file 2 [file Data_Sheet_1.pdf]
